# Nurse in limbo: A qualitative study of nursing in disasters in Iranian context

**DOI:** 10.1371/journal.pone.0181314

**Published:** 2017-07-31

**Authors:** Negar Pourvakhshoori, Kian Norouzi, Fazlollah Ahmadi, Mohammadali Hosseini, Hamidreza Khankeh

**Affiliations:** 1 Health in Emergency and Disaster Research Center, University of Social Welfare & Rehabilitation Sciences, Tehran, Iran; 2 Department of Nursing, University of Social Welfare & Rehabilitation Sciences, Tehran, Iran; 3 Department of Nursing, Faculty of Medical sciences, Tarbiat Modares University, Tehran, Iran; 4 Department of Nursing and Health in Emergency and Disaster Research Center, University of Social Welfare & Rehabilitation Sciences, Tehran, Iran; 5 Health in Emergency and Disaster Research Center, University Of Social Welfare & Rehabilitation Sciences, Tehran, Iran, Department of Clinical Science and Education, Karolinska Institute, Stockholm, Sweden; University of South Australia, AUSTRALIA

## Abstract

**Background:**

An understanding of nurses’ experiences in disasters can help to identify their problems in this area. These can be overcome with better planning and preparation. The aim of this study was to explore the experiences and perceptions of disaster nurses regarding their provision of disaster health care services.

**Methods:**

This was a qualitative study using an inductive qualitative content analysis. Participants included 15 Iranian nurses who had experiences of health care delivery in disasters. A purposeful sampling was applied until data saturation was reached. Data were collected using semi-structured interviews and then analyzed based on the principle of inductive content analysis.

**Results:**

Five main categories emerged from the experiences and perceptions of nurses who were involved in providing health care services in disasters: afraid of probability of recurrence, necessity of providing healthcare services for an unknown period of time, challenge of what to prioritize, nurses’ own conflicting emotions, and their concern for their own families.

**Discussion:**

There are several factors affecting the delivery of healthcare in disasters. Nurses, who feel better prepared and have some understanding of the ethical implications of working under different standards of care, may be more comfortable with care giving in disasters. Appropriately, training and preparing nurses for disasters is important for optimizing the safe functioning and minimizing emotional and psychological damage.

## 1. Introduction

Considering that natural disasters compromise social health and welfare, providing affordable health care is one of the main factors influencing survival, reduced mortality, and increased welfare of the people after the occurrence of such incidents[1–3]. In such situations, a quick and effective health response is needed beyond the usual emergency response [4–6].

Nurses play a crucial active role in managing disaster situations. Their caring role causes community members to trust them and they have the expertise to deliver clinical care, team leadership, creative problem-solving skills, resource management, as well as having important communication skills in situations of rapid changes[7].

According to some studies, nurses who work in disasters experience many problems such as safety concerns [3,8], stress and fatigue [8], and ethical challenges [8–10]. Nurses in disaster situations, must be sufficiently prepared for the situation, and they always have more problems and challenges in their work [11].

Some studies have shown that the problems nurses are passing through during disasters include psychological problems such as stress from work, health problems, family problems, organizational problems, poor coordination, and problems such as low awareness of people [9,12,13]. Five issues regarding working in disaster situations have been explored by Wenji (2014) including insurmountable challenges, the qualities of a disaster nurse, mental health and trauma, poor disaster planning and co-ordination, and urgently needed disaster education[10].

While Iran experiences significant disasters annually[14,15], there are limited studies in the field of disaster nursing that explore their problems[16–18]. Uncovering nurses’ experiences regarding disaster relief is important to inform future relief efforts and the development of programs to enable nurses participate with some confidence in future events[19].

Since process of providing care in disasters is a phenomenon that consists human relationships, action-interactions, and emotions that could not be assessed by quantitative approaches, qualitative method was selected as an approach. By qualitative approaches, we can observe phenomenon from the eyes of the participants, and the hidden aspects of phenomenon can be explored[20]. Therefore, the researchers decided to conduct a study to explore the experiences and perceptions of nurses who have served in times of disaster in Iran in order to identify their real problems and challenges. In this way, a clearer picture of the experiences and difficulties faced by nurses while providing care in special situations, such as disasters, is available as a basis for planning and policy-making in the health care system, to provide appropriate quality service and ultimately decrease the people’s suffering.

## 2. Materials and methods

An inductive qualitative content analysis approach was used to analyze the data to attain a condensed and broad description of nurses’ experiences; the analysis outcomes were concepts or categories describing their experiences. In the present study, data were collected without assuming any previous hypothesis. The acquired data were based on the participants’ view. Codes and categories were obtained using the inductive process, conceptually ordered considering properties and dimensions were developed [21–23].

### 2.1. Participants and setting

The 15 participants were selected purposefully and interviewed in order to collect and generate rich data. They were all nurses who had experience in providing healthcare services at the time of natural disasters including nurses who had a managerial role at that time, university professors, and specialists in the field of natural disasters. To achieve the maximum variability, we tried to select nurses who have served in various natural disasters and also participated in various positions. Unwillingness to participate during the study was considered as exclusion criterion.

### 2.2. Data collection and analysis

The present qualitative study used content analysis as the research method for subjective interpretation of the interviews’ content, through a systematic classification of coding and identifying the concepts or patterns. The selection of participants was determined utilizing a purposive sampling method[20]. The participants were included if they had previous experience regarding healthcare in disasters. The subjects had various natural disasters experiences and also various positions. Fifteen interviews were conducted with experts and managers of disasters including 7 Nursing manager, and 8 nurses. Participants were within the age range of 34–51 years, with a mean age of 40.87 ± 5.14 years, and a mean working history of 15.4 ± 5.2 years (Table 1).

**Table 1:** Participant characteristics.

No	Age (years)	Position in disaster	Working history (years)
1	45	Nurse, Nursing manager	18
2	43	Nurse, Nursing manager	18
3	43	Nurse, Nursing manager	22
4	34	Nurse	8
5	51	Nurse	23
6	42	Nurse	13
7	43	Nurse, Nursing manager	17
8	44	Nurse, Nursing manager	16
9	34	Nurse	11
10	35	Nurse	9
11	34	Nurse	10
12	37	Nurse	12
13	39	Nurse	11
14	46	Nurse, Nursing manager	20
15	43	Nurse, Nursing manager	23

Semi-structured interviews were utilized as the research instrument. Purposive sampling continued until the saturation point of each concept was reached, and further data collection failed to provide additional information; the sample size was determined by data saturation. The researcher continued to gather data until reaching the level of data saturation. Saturation is usually explained in terms of “when no new categories or relevant themes are emerging”. But saturation is more than a matter of no new categories or themes emerging. It is also denotes a development of categories in terms of their properties and dimensions, including variation, and possible relationships to other concepts. In other words, the aim of research is not just to come up with a list of categories. It is to tell us something about those categories. The understandings provided by the researcher must go beneath surface explanations[20]. On average, each interview lasted between 30 and 61 min, and they were conducted in Persian by the same interviewer; they were transcribed verbatim, and then translated into English. The content analysis was performed on the data written in Persian, before translation. The interview guide included a short list of general questions. This was used as a tool for initiating the interviews. During each interview, more specific questions were asked. Examples of the questions are: “What problems do you face during healthcare in disasters?” and “In the face of disasters what do you do?” In addition, complementary questions were added when necessary: ‘‘could you elaborate more on your experience?”

During the open coding phase, all of the interview texts were read several times, and the keywords, phrases, incidents, and facts were noted. The primary codes were extracted, and the codes and data were compared in order to find similarities and differences; afterwards, the categories and sub-categories were developed. A preliminary set of codes, categories, and sub-categories were formed from the first interview, and the emerging codes were considered as the results [21–23].

Considering the methodology of content analysis [21,22], the interviews were carried out by the same interviewer.

### 2.3. Ethical considerations

The procedures used in this study caused no physical or psychological harm to the participants. The Ethics Committee of The University of Social Welfare and Rehabilitation Sciences approved the study and examined ethical considerations. All participants were informed about the purpose of the research, they were guaranteed that their names would remain confidential in any reports of the study and then recording of the interviews followed. The subjects had the right to withdraw from the study at any time. Written informed consent was provided for all participants.

### 2.4. Rigor

Analysis of the interviews was conducted using the Lundmen and Grancheim five stages content analysis method [22]. Four criteria of creditability, transferability, dependability and confirmability achieved were used to provide the validity and reliability of results of this study [24]. To ensure that the analysis disclose nurses’ experiences, member check was done during the data collection, and some changes were made if needed. For creditability of the findings, we presented the quotes of participants, for better perceptions of readers about the study results. The results of the analyses, such as the codes and subcategories were checked by some experts for dependability and confirmability. The external check method was applied using two authors (the first and corresponding authors) expert in disasters and after that peer-checked was carried out by two PhD students who had previous experience in the field of study. In addition, the transferability of data was confirmed by maximum variation of sampling.

## 3. Results

Study participants were 15 nurses who had experience providing nursing intervention after a disaster, expertise in the field of disaster nursing and experience as a manager following recent Iranian disaster events. After data analysis, five main categories were extracted from the data: Afraid of probability of reccurence, necessity of providing healthcare services for an unknown period of time, challenge of what to prioritize, nurses’ own conflicting emotions, and their concern for their own families (Table 2). These categories with their subcategories have been explained below.

**Table 2:** Examples of extracting of codes, sub categories, and categories from raw data.

Meaning unit	Code	Subcategory	Category
After a few days I came to city, oh, what the situation is and it was very busy and yet people got the shock even though my family was, fortunately, did not happen to them but all of them still had the fear(p5)	Fear following the incident	Fear of the likelihood of recurrence of an accident	Afraid of probability of reccurence
	Anxiety because of the probability of this event		
Lack of sleep, because I’m anxious, because there were regular aftershocks(p14)	Insomnia followed by anxiety, associated with the probability of this event	Lack of rest, followed by fear of recurrence of event	
	Nurses continuity of service delivery	Physical fatigue and illness after providing healthcare continuously	The necessity of providing healthcare services for an unknown period of time
Fatigue itself was a big issue. Nurses were in the hospital for a long time and there were no shifts, all staff that stayed in the hospital after 48 hours, almost all of them were in trouble, even 2–3 people were admitted there(p9)	Fatigue and illness following personnel continuity in the provision of services		
In the first few days of the disaster, everything is chaotic, but health care delivery is not just for one day, sometimes you have to be present at the disaster zone for one, two or even three months(p1)	The need for long-term presence at the scene of disaster	Long-term presence on the scene of disaster	
	The need for continuity of service at the time of disaster		
The situation is very different in the disaster zone, limited resources, shortage of manpower, you are not able to provide all services during a disaster, and you choose who to give care to. The ethical debate is happening(p8)	Nurse ethical challenges followed by a lack of resources and manpower in disaster	Ethical challenges followed by a lack of power and resources	Nurses’ challenge of what to priortize
A major part of what we saw were ethical issues. We actually did not see anything else other than this(p6)	High ethical challenges in service delivery in the disaster		
	The challenge for nurses in choosing what to prioritize in a disaster	Nurses’ moral challenge -choosing who to give care to	
At the time of disaster you will choose who to give care to.The ethical debate always is present(p1)	Nurse ethical challenges in service at the time of disaster		
	Nurses ethical challenges due to the lack of service delivery program in disasters	The absence of plans to service delivery: an ethical challenge for nurses	
There was no plan. You have to decide what to do. Certainly you always were debating with yourself about doing the right thing(p14)	Lack of service delivery programs: factor for ethical challenges the nurse		
I wanted to do something but I was not able to do it, so I feel guilty, I was confused and I was feeling really bad(p13)	Contradictory feelings/nurses emotions duality	Nurses’ own conflicting emotions because of lack of resources	Nurses’ own conflicting emotions
	nurses contradictory feelings due to lack of facilities		
	Nurses contradictory feelings due to inability to provide services	Nurse trying barren despite the desire to help	
We went as a volunteer helper,but in fact, we can do anything,there was no one to remove the injured from the rubble so that we can offer care (p12)	Inability to be effective in the scenes in disaster, causing emotional conflict		
All were involved in some way, nurses and officials were involved with their families(p14)	Concern for their family, the biggest concern of the nurses at a time of disaster	Worry for the families is nurses’ first concern in disasters	Nurses concern for their own families
I was present at some event, the first concern of nurses were their families, it was important for them(p8)	Worry for the families is the primary concern of nurses in disasters followed by the inability to communicate with them		

### 3.1. Afraid of probability of reccurence

In this study, participants revealed that their anxiety and fear regarding the possible recurrence of this event could affect their service delivery. This category had two subcategories: the fear of the possible injury to themselves following disasters, and a lack of adequate opportunity for rest, due to fear of recurrence of the event. Most participants had experienced a lot of fear and anxiety regarding the probable recurrence of this event. Participant 5 said:

“After a few days I came to the city, oh, the situation! And it was very busy and yet people got the shock even though, fortunately, my family was safe, but all of them were still scared”

On the other hand, due to the likelihood of recurrence, nurses had a risk of injury to themselves. This affected their ability to rest and, additionally, due to the staff limitations, there was limited time to rest and to restore their energy to be prepared for work the next day.

Participants preferred to be woken regardless of their exhaustion. In this regard, Participant 9 said the following:

“We have an hour to lie down and rest, but there was an aftershock and a part of the airport ceiling was hanging, every moment we thought the roof might be destroyed over our heads. Even the landing of the aircraft caused severe shocks. After a while, we found we could not rest”

### 3.2. Necessity of providing healthcare services for an unknown period of time

Due to the special circumstances of disasters, nurses providing healthcare did not know when they would be able to return to their houses and this uncertainty was disturbing for them. This category had two subcategories including fatigue and illness after providing long term care, and long-term exposure to the scene of a disaster. Many of participants stated that long-term work had a negative impact on their physical condition, in some cases leading to illness and hospitalization of the nurses. In this regard, Participant 3 said:

“We had 10 consecutive days working in the hospital and then I was in hospital for 10 days due to severe fatigue. A lot of nurses were sick, including me. I was hospitalized again for 10 days due to high work pressure.”

The second subcategory was long-term presence at the scene of the disaster. According to the results of this study and due to disaster situations, almost all team members have to provide long and continuous health care services. Often, due to lack of nursing staff, there were no relief staff to fill nurses’ shifts so they had to work continually for a long period of time to provide services. Based on this, Participant 1 said:

“In the first few days of the disaster, everything is chaotic, but health care delivery is not just for one day, sometimes you have to be present at the site of the disaster for one, two, or even three months.”

### 3.3. Nurses’ challenge of what to prioritize

The experiences of the participants indicated that, in disasters, they were often forced to choose between different options. Therefore, they always faced ethical challenges, and this is the constant concern for them–did I chose the right option or not? This category had three subcategories: ethical challenges due to shortages of staff and resources, the nurses’ moral challenge of having to decide what to prioritize, and the further ethical challenge of what to do in the absence of plans to provide nursing service. In relation to the first subcategory, participants expressed that, since in disaster situations there is always a shortage of nursing staff and lack of resources, nurses should always choose the right thing to do. Participant 8 said:

“The situation is very different in the disaster, limited resources, shortage of staff, you are not able to provide all services during a disaster, and you choose who will be the first priority to be cared for. The ethical debate is happening.”

In relation to the second subcategory, nurses’ moral challenge regarding what to prioritize, participants believed that, finding priority among the victims was difficult for them. Participant 2 said:

“Sometimes you may encounter your relatives among victims, which is where the judgment and ethical debate comes. You ask yourself, now should I have to choose my family or others? A nurse told his experience of the earthquake in Azerbaijan that my son and my brother went to the hospital at the same time, and I did not know what to do? Which of them should I take care of first?”

In relation to the third subcategory, the absence of plans to provide service, participants stated that since there was no clear plan for service delivery, many factors had an impact including the insistence of the victims’ families. Nurses were challenged and had to provide services to victims who were with their families and those who were alone were at the end of line of service delivery. Participant 3 said:

“You know? Because the victims’ families pulled us to this side or that side, on the other hand, there was no plan. The priority of care delivery depends on who cries more, to attract the nurses’ attention to attend to their victims. Everyone tried to show that their patients were in more urgent need compared to the other patients.”

### 3.4. Nurses’ own conflicting emotions

Nurses during disasters often experience mixed feelings; and sometimes they were in conflict with their emotions. This category had two subcategories: nurses’ emotional conflict due to lack of resources, and nurses’ inability to provide assistance despite their desire to help. In relation with first subcategory, Participant 13 said:

“I wanted to do something but I was not able to do it, so I feel guilty, I was confused and I was feeling really bad.”

Regarding the second subcategory, participants talked about their energy and passion to help; however, due to undesirable conditions, their presence seemed ineffective so they had feelings of helplessness and despair. Participant 9 said:

“I always had a guilty conscience, we had a lot of energy, but could not do anything well, and I do not know… perhaps the most uncomfortable thing for us was that we did not have the ability to remove debris while perhaps someone was still alive under the rubble, what can we do?”

### 3.5. Nurses’ concern for their own families

For almost all participants in this study, nurses’ primary concern was for their families, and it is always on their minds. Some participants described it as a reason for nurses failing to focus on the service delivery. Participant 14 said:

“All were involved in some way, nurses and officials were involved with their families.”

Participant 5 said:

“All of us were worried about our houses; because of the shortage of nurses we had to stay in the hospital, but we were constantly worried, the telephones were disconnected so we could not receive any news from our family.”

## 4. Discussion

In this study, the five categories explored included the Afraid of probability of reccurence necessity of providing health care services for an unknown period of time, challenge of what to prioritize, nurses’ own conflicting emotions, and their concern for their own families. These concepts illustrate the problems nurses experience at the time of disasters to provide health care services for affected population. Some of categories include specific sub-categories, based on their distinct properties in a given category.

According to the results of this study, the first problem that nurses experience at the time of disaster was being worried about the possibility of event recurrence. In line with the results of this study, Schlenger & Jernigan (2003) described nurses emotions resulting from working in disaster situations to include fear, sorrow, uncertainty, and anger; and, clinically significant results may include PTSD and ASD[25].

In addition, O’Boyle (2006) discussed that nurses indicated they believe working during a bioterrorism event would be chaotic and frightening. Staff issues were also concerned about this [26]. Furthermore, Holloway et al. (1997) reported how the physical and safety needs of disaster workers will increase nurses’ workload and contribute to increased psychological distress [27]. Due to uncertainty in a disaster situation, nurses have a lot of stress, which should be reduced; also, Chang et al. (2006) in their study concluded that reduction of workplace stress and increase support for nurses can strengthen their mental health [28].

In this regard, exercise increase the tolerance level of individuals from the burden of disasters, especially in events like earthquakes[15]. Fu-jin (2002) reported that service providers after seeing severe destruction and experiencing a lack of control over the situation and the probability of the event, had severe fear and nightmares. These issues show the need to follow up nurses who have served in disasters on mental health problems[8].

In addition, Nekooei Moghaddam (2014) reported mental and psychological stress experienced by both traumatized people and caregivers. The anxiety of some caregivers has affected their performance[29].

Other studies have concluded that education and preparation of nurses is necessary to decrease emotional and psychological stress [26,30], and that nurses have to know how to reduce their stress [2]. Based on this, Chapman et al. (2008) reported that stress control for caregivers is one of the most important issues that should be taught in disaster workshops. They should also have access to consultation services [31].

The necessity of providing healthcare services for an unknown period of time was another problem that emerged in this study. Pre-hospital emergency care has specific problems because of the special conditions that increase the difficulties of working in this area.

The following study, though not in the same area, has some similarities to the results of this study; although the study was not exactly the point, but similarities can be seen in the results of some studies. For example in the study of Saberi Nia (2013), one of the important problems nurses in pre-hospital sites face is physical health problems resulting from factors such as the number of missions, as well as, there is a need for strong mental health in addition to the physical health of the workers. [13].

Furthermore, Wenji et al. (2015) reported that lack of disaster nursing experience was a major problem; and nurses had to be committed and try to be in good physical and psychological health to work competently. Ability to deal with environmental conditions was very important. Nurses said passing long distances to the disaster fields required patience and endurance and when they arrived they were too tired [10].

In this regard, Sebastian et al (2003) reported nurses experienced concerns about conditions of working in disasters, such as rest, and reported being on duty 24 h a day[32]. In addition, Pesik et al. (2001) stated that during a bioterrorism event, there may be an increased demand for infrequently used supplies, which could lead to a shortage of critically needed resources. Resources, including staff, can be overwhelmed by the large number of people seeking help with acute injuries, as well as the large number of people who may present demanding prophylactic treatment [33].

Nurses’ challenges regarding what to prioritize were another category that emerged in this study. Chinese nurses experiences in Wenji et al. (2015) revealed that participants tried to put personal feelings aside during their work, but faced ethical dilemmas when giving care[10].

It seems ethical issues that have been mentioned in different cultures are different.The moral conflict between professional ethics and survival by participants was discussed as giving care at the time of aftershocks, where personal safety was raised. Stay or flee in the face of hazard, was a matter of professional and ethical issues [34].

Unlike soldiers, firefighters and police officers, health care workers do not receive any training in hazardous environments and work under different professional and ethical dilemmas. Although they carry out their duties seriously, some participants had found themselves in situations full of moral challenges and they were not trained to deal with such situations. Unfortunately no attention has been paid to the moral preparedness of people who are at the forefront of service delivery in disasters [35].

In line with this, Aliakbari et al. (2015) in their study on ethical challenges associated with disaster nursing in Iran noted that one of sub-themes in this field was professional ethics that highlights how a disaster changes and challenges everyday nursing routine and practice. Despite this, nurses felt a professional obligation and responsibility that extends beyond their individual needs. It was observed that despite the conditions, nurses focused on professional and ethical responsibility. All nurses affirmed the importance of commitment and responsibility in their profession. They believed that disaster puts nurses in place beyond the normal scope of their activities [16].

Nurses’ own conflicting emotions was another category that emerged in this study. This important concept was not mentioned in other studies, but results of this kind have been seen in some studies. For example, Wenji et al. (2015) argued about how nurses are faced with many challenges about leaving injured elderly who refused to leave their homes being destroyed [10].

In addition, Riba & Reches (2002) reported Israeli nurses’ experiences and showed that they separated themselves entirely from their emotions. Their thoughts and feelings remained in the background just when he was feeling the harsh conditions they were shown. As soon as the last victim was treated, nurses start to reveal their feelings [36].

In almost all related studies, the first mental and primary concern of nurses was the safety of their families. Nekooei Moghaddam (2014) reported that nurses had said during their work they need to have confidence that their families have been supported[29]. In addition, French et al. (2002) noted that the nurses had experienced a conflict between family and work commitments, especially when they are simultaneously victims and caregivers. In response to Hurricane Floyd, nurses preferred to be with their families and had a lot of anxiety when they were forced away from their family members. Even some of them lost their jobs when they decided to stay with their families[37].

Moreover, in another study, nurses expressed concern over their personal safety, their family’s safety, family commitments, insufficient food, water, and resting facilities [26]. Communication with family was a particular concern during periods of quarantine or extended shifts [38,39]. Actually, communication with family members is a very important issue expressed by Australian aid workers and they suggested the creation of communication channels preset and strategies for contact with their loved ones could increase their willingness to participate in work at the time of incidents [39].

## 5. Conclusion

This study showed that various factors affect nurses when they provide health care services in disasters. In our study, five main categories emerged, which were the greatest concerns of disaster nurses. Often in disasters, nurses are expected to act in chaotic workplaces and provide direct care to patients affected by disasters. Stress in this situation will be heightened when nurses feel anxiety and fear for themselves and their families. Results revealed that nurses who feel better prepared, and had some understanding of moral implications of working under different standards of care, may be better suited for health care delivery in disasters. Those who had no choice about going to the disaster area or had less choice are more likely to have feelings such as lack of usefulness. In addition, the nurses’ training and preparation for disasters is essential to optimize the safe functioning and minimize the emotional and psychological trauma. Therefore, to explore the process of nursing preparedness further qualitative research, using a grounded theory, is recommended.

## Supporting information

S1 Dataset Parts of data. (RAR)

S1 Appendix PC_interview_oral history(1)-Consent form for qualitative. The consent form of the study.
(DOC)

S2 Appendix Certification letter from native English Edit. (PDF)

S3 Appendix Consolidated criteria for reporting qualitative studies. (DOC)
