# Supplementary material for: Nurse in limbo: A qualitative study of nursing in disasters in Iranian context
Source: PLoS One. 2017 Jul 31;12(7):e0181314. doi: 10.1371/journal.pone.0181314 (PMC5536275; doi:10.1371/journal.pone.0181314)
Supplement: S1 Appendix — The consent form of the study. (DOC) [file pone.0181314.s002.doc]

**Nurse in limbo: A qualitative study of disaster nursing in Iran**

**PARTICIPANT CONSENT FORM**

I, ................................................................................... [NAME], agree to take part in this research study.

In giving my consent I state that:

- I understand the purpose of the study, what I will be asked to do, and any risks/benefits involved.
- I have read the Participant Information Statement and have been able to discuss my involvement in the study with the researchers if I wished to do so.
- The researchers have answered any questions that I had about the study and I am happy with the answers.
- I understand that being in this study is completely voluntary and I do not have to take part. My decision whether to be in the study will not affect my relationship with the researchers or anyone else at the University of Social Welfare and Rehabilitation Sciences now or in the future.
- I understand that I can withdraw from the study at any time.
- I understand that I may stop the interview at any time if I do not wish to continue, and that unless I indicate otherwise any recordings will then be erased and the information provided will not be included in the study. I also understand that I may refuse to answer any questions I don’t wish to answer.
- I understand that personal information about me that is collected over the course of this project will be stored securely and will only be used for purposes that I have agreed to. I understand that information about me will only be told to others with my permission, except as required by law.
- I understand that the results of this study may be published, but these publications will not contain my name or any identifiable information about me

I consent to:

- **Audio-recording** YES 🞏 NO 🞏
- **Being contacted about future studies** YES 🞏 NO 🞏
- **Reviewing transcripts** YES 🞏 NO 🞏

**Would you like to receive feedback about the overall results of this study?**

**YES** 🞏 **NO** 🞏

If you answered **YES**, please indicate your preferred form of feedback and address:

🞏 Postal: _______________________________________________________

___________________________________________________

🞏 Email: ___________________________________________________

.................................................................

**Signature**

....................................................

**PRINT name**

..................................................................................

**Date**
