# Supplementary material for: Nurse in limbo: A qualitative study of nursing in disasters in Iranian context
Source: PLoS One. 2017 Jul 31;12(7):e0181314. doi: 10.1371/journal.pone.0181314 (PMC5536275; doi:10.1371/journal.pone.0181314)
Supplement: S2 Appendix — (PDF) [file pone.0181314.s003.pdf]

EDITORIAL CERTIFICATE LETTER

This letter is to certify that this organization employs only native English-speaking citizens of English-speaking countries as editors of academic articles. **EnglishEdit.ir** certifies that the text of the article mentioned below has been edited by a native English speaker with appropriate experience and qualifications for proper English language, grammar, punctuation, spelling, and overall style.

The substantive content of the article mentioned below remains the full responsibility of the author/authors:

TITLE OF ARTICLE:

NURSE IN LIMBO: A QUALITATIVE STUDY OF DISASTER NURSING IN IRAN

AUTHOR(S):
NEGAR, POURVAKHSHOORI .PHD CANDIDATE, KIAN, NOROUZI. PHD , FAZLOLLAH, AHMADI . PHD , MOHAMMADALI, HOSSEINI. PHD , HAMIDREZA, KHANKEH . PHD

Radan English Edit
WWW.englishedit.ir
Tel: 021-22362401

B. Radan
Radan English Edit
Head of Institute

This certificate may be verified at www.Radanenglishedit.com
Documents receiving this certification should be English-ready for publication; however, the author has the ability to accept or reject our suggestions and changes.
www.Radanenglishedit.com ; englishedit.ir@gmail.com
No. 10, Shabnam St., Jamshidi St. Saadat Abad, Tehran, Iran
Tel: +98 -21-22362401; 0910-201-3545
