# Supplementary material for: Nurse in limbo: A qualitative study of nursing in disasters in Iranian context
Source: PLoS One. 2017 Jul 31;12(7):e0181314. doi: 10.1371/journal.pone.0181314 (PMC5536275; doi:10.1371/journal.pone.0181314)
Supplement: S3 Appendix — (DOC) [file pone.0181314.s004.doc]

Consolidated criteria for reporting qualitative studies (COREQ): 32-item checklist

| **No** | **Item** | **Guide questions/description** |
| --- | --- | --- |
| **Domain 1: Research team and reflexivity** |  |  |
| Personal Characteristics |  |  |
| 1. | Interviewer/facilitator | Which author/s conducted the interview or focus group? The first author |
| 2. | Credentials | What were the researcher's credentials? PhD, Post-doc researcher |
| 3. | Occupation | What was their occupation at the time of the study? PhD , Lecturer |
| 4. | Gender | Was the researcher male or female? female |
| 5. | Experience and training | What experience or training did the researcher have? She passed the courses and workshops about that. |
| Relationship with participants |  |  |
| 6. | Relationship established | Was a relationship established prior to study commencement? Before beginning the interviews the interviewer introduced her and if they like to participate in research she explained about study goals and their privacy. |
| 7. | Participant knowledge of the interviewer | What did the participants know about the researcher? The researcher explained about personal goals, reasons of doing study, study goals and the participants’ privacy. Furthermore about if they like they can leave the study at any time. |
| 8. | Interviewer characteristics | What characteristics were reported about the interviewer/facilitator? She had interest and motivation for doing the study. She passed many educational and training courses for interview and doing qualitative study. She was hardworking and with great perseverance without any bias. |
| **Domain 2: study design** |  |  |
| Theoretical framework |  |  |
| 9. | Methodological orientation and Theory | What methodological orientation was stated to underpin the study? Content analysis |
| Participant selection |  |  |
| 10. | Sampling | How were participants selected? purposive and snowball |
| 11. | Method of approach | How were participants approached? Face to face |
| 12. | Sample size | How many participants were in the study? 15 |
| 13. | Non-participation | How many people refused to participate or dropped out? Reasons? 2 people for their bad memories about disasters they didn’t want to recall the experiences. |
| Setting |  |  |
| 14. | Setting of data collection | Where was the data collected? Interviews lasted each interview lasted between 30 and 61 min. |
| 15. | Presence of non-participants | Was anyone else present besides the participants and researchers? No |
| 16. | Description of sample | What are the important characteristics of the sample? Participants were included nurses who had experience providing healthcare at the time of disasters; nurses who had a managerial role at the time of disasters; and university professors and specialists in the field of natural disasters. |
| Data collection |  |  |
| 17. | Interview guide | Were questions, prompts, guides provided by the authors? Was it pilot tested? To collect data, semi-structured interviews were begun with an open question according to main research question. And gradually according to data analysis, in-depth inquiry and probe questions about nurses' experiences of providing healthcare at the time of the disasters continued.  The interview guide included a short list of general questions. This was used as a tool for initiating the interviews. During each interview, more specific questions were asked. Examples of the questions are: “What problems do you face during healthcare in disasters?” and “In the face of disasters what do you do?” In addition, complementary questions were added when necessary: ‘‘could you elaborate more on your experience?’’ |
| 18. | Repeat interviews | Were repeat interviews carried out? If yes, how many? One interview repeated. |
| 19. | Audio/visual recording | Did the research use audio or visual recording to collect the data? Yes. Voice recorder was used. |
| 20. | Field notes | Were field notes made during and/or after the interview or focus group? Yes. |
| 21. | Duration | What was the duration of the interviews or focus group? Interviews lasted between 30 and 61 min. |
| 22. | Data saturation | Was data saturation discussed? Yes. |
| 23. | Transcripts returned | Were transcripts returned to participants for comment and/or correction? Yes. |
| **Domain 3: analysis and findings** |  |  |
| Data analysis |  |  |
| 24. | Number of data coders | How many data coders coded the data? Data coded by first author and codes checked and revised by corresponding author in many times. |
| 25. | Description of the coding tree | Did authors provide a description of the coding tree? Yes. |
| 26. | Derivation of themes | Were themes identified in advance or derived from the data? From the data. After review the codes with texts(review, compare the similarities and differences between codes and the combination of the similar codes) ,classification and development of categories based on similarities and differences, review categories and compare this with the data to ensure the strength of codes, identify themes with depth reflection and compare the categories and finally, the findings were reported. A subset of sub-categories, categories and codes from the first interview were formed and finally emerged codes were considered.  During the open coding phase, all of the interview texts were read several times, and the keywords, phrases, incidents, and facts were noted. The primary codes were extracted, and the codes and data were compared in order to find similarities and differences; afterwards, the categories and sub-categories were developed. A preliminary set of codes, categories, and sub-categories were formed from the first interview, and the emerging codes were considered as the results. |
| 27. | Software | What software, if applicable, was used to manage the data? No. |
| 28. | Participant checking | Did participants provide feedback on the findings? Yes. |
| Reporting |  |  |
| 29. | Quotations presented | Were participant quotations presented to illustrate the themes / findings? Was each quotation identified? Yes. |
| 30. | Data and findings consistent | Was there consistency between the data presented and the findings? Yes. |
| 31. | Clarity of major themes | Were major themes clearly presented in the findings? Yes. |
| 32. | Clarity of minor themes | Is there a description of diverse cases or discussion of minor themes? Yes. |
